# Genome-Wide Single-Nucleotide Polymorphisms Discovery and High-Density Genetic Map Construction in Cauliflower Using Specific-Locus Amplified Fragment Sequencing

**DOI:** 10.3389/fpls.2016.00334

**Published:** 2016-03-21

**Authors:** Zhenqing Zhao, Honghui Gu, Xiaoguang Sheng, Huifang Yu, Jiansheng Wang, Long Huang, Dan Wang

**Affiliations:** ^1^Institute of Vegetables, Zhejiang Academy of Agricultural SciencesHangzhou, China; ^2^Biomarker Technologies CorporationBeijing, China

**Keywords:** cauliflower, SLAF, SNP, sequencing, genetic map

## Abstract

Molecular markers and genetic maps play an important role in plant genomics and breeding studies. Cauliflower is an important and distinctive vegetable; however, very few molecular resources have been reported for this species. In this study, a novel, specific-locus amplified fragment (SLAF) sequencing strategy was employed for large-scale single nucleotide polymorphism (SNP) discovery and high-density genetic map construction in a double-haploid, segregating population of cauliflower. A total of 12.47 Gb raw data containing 77.92 M pair-end reads were obtained after processing and 6815 polymorphic SLAFs between the two parents were detected. The average sequencing depths reached 52.66-fold for the female parent and 49.35-fold for the male parent. Subsequently, these polymorphic SLAFs were used to genotype the population and further filtered based on several criteria to construct a genetic linkage map of cauliflower. Finally, 1776 high-quality SLAF markers, including 2741 SNPs, constituted the linkage map with average data integrity of 95.68%. The final map spanned a total genetic length of 890.01 cM with an average marker interval of 0.50 cM, and covered 364.9 Mb of the reference genome. The markers and genetic map developed in this study could provide an important foundation not only for comparative genomics studies within *Brassica oleracea* species but also for quantitative trait loci identification and molecular breeding of cauliflower.

## Introduction

Cauliflower (*Brassica oleracea* var. *botrytis*, 2n = 2x = 18) is an important vegetable crop worldwide. It is considered a vital source of vitamins, dietary fiber, antioxidants, and anti-carcinogenic compounds (Volden et al., 2009; Picchi et al., 2012). In 2013, global cauliflower cultivation had spread across ~1.2 million hectares, with total production of ~20.9 million metric tons (http://faostat.fao.org/). Due to the economic importance and nutritional value of cauliflower, great efforts have been taken to improve its yield and quality. Nevertheless, breeding methods used are essentially conventional and less-effective, resulting in relative slow progress of the cauliflower breeding program (Gu et al., 2014). Modern strategies such as marker-assisted selection (MAS) are therefore necessary to accelerate cauliflower genetic improvement.

Molecular markers and genetic maps are considered an important foundation for quantitative trait loci (QTL) mapping and MAS (Kato et al., 2014; Li et al., 2014; Wu et al., 2014; Zhang et al., 2014). As one of the subspecies of *B. oleracea*, cauliflower has been employed to develop a series of segregating populations to construct several C-genome genetic maps, by crossing with other subspecies of *B. oleracea*, including kale (*B. oleracea* var. *acephala*) (Kianian and Quiros, 1992), broccoli (*B. oleracea* var. *italica*) (Li et al., 2003; Gao et al., 2007), collard (*B. oleracea* var. *acephala*) (Hu et al., 1998), and brussel sprouts (*B. oleracea* var. *gemmifera*) (Sebastian et al., 2000, 2002), and to identify several QTLs involved in common traits such as flowering time (Kianian and Quiros, 1992), glucosinolate profile (Gao et al., 2007), and leaf traits (Sebastian et al., 2002). However, because of the organ specificity of cauliflower curd, inter-subspecies populations are unsuitable for QTL analysis of many curd-specific traits, which are most important for cauliflower breeding. Hence, a cauliflower × cauliflower based population has extensive potential for marker discovery, genetic mapping, and QTL analysis.

Several molecular markers such as restriction fragment length polymorphism (RFLP), randomly-amplified polymorphic DNA (RAPD), amplified fragment length polymorphisms (AFLP), sequence-related amplified polymorphism (SRAP), and simple sequence repeats (SSR) have been widely used in plant genetic research. However, the utilization of these markers has always been limited by the higher time and cost requirements as well as limited marker resource (Zhao et al., 2014a). The advent of massive, parallel, next-generation sequencing (NGS) technologies have accelerated and simplified the identification of sequence variants, enabling large-scale single-nucleotide polymorphism (SNP) discovery throughout the genome (Zhou et al., 2014). Considering that whole-genome deep re-sequencing is still expensive and usually not necessary (Davey et al., 2011; Wei et al., 2014), several simplified and cost-effective methods for SNP discovery and high-throughput genotyping have been developed, such as reduced representation library (RRL) sequencing (Van Tassell et al., 2008), restriction-site associated DNA sequencing (RAD-seq; Miller et al., 2007), two-enzyme genotyping by sequencing (GBS) (Poland et al., 2012), and sequence-based genotyping (SBG) (Truong et al., 2012). More recently, specific-locus amplified fragments sequencing (SLAF-seq) was developed as a streamlined RRL sequencing approach for high-resolution de novo SNP discovery and genotyping (Sun X. et al., 2013). A high density genetic map including 1233 high-quality markers has been developed using this strategy on a sesame F_2_ population (Zhang et al., 2013). This study showed that SLAF sequencing was a powerful high-throughput technique for plant genome research. To date, this strategy has also been successfully applied in several other species including soybean (Li et al., 2014; Qi et al., 2014), cucumber (Wei et al., 2014; Xu et al., 2014), tea plant (Ma et al., 2015), and grape (Guo et al., 2015).

In this study, we generated a double-haploid (DH) population derived from a cross between two different types of cauliflower common in production, including an advanced inbred line of traditional compact-curd cauliflower and a DH line of loose-curd cauliflower. Based on this cauliflower × cauliflower population, SLAF-seq was then employed to detect large-scale SNPs and construct a high-density genetic map that could be used to provide a platform for future QTL mapping and MAS.

## Materials and methods

### Mapping population development and genomic DNA isolation

An advanced inbred line of compact-curd cauliflower “4305” (F_8_) and a DH line of loose-curd cauliflower “ZN198” were used to develop the DH mapping population (Figure S1). There are significant morphological differences between the two homozygous lines, especially for several agronomically important traits including curd size, curd weight, curd shape, and curding-time. A microspore culture protocol as previously described by Gu et al. (2014) was used to produce regenerated plants from a single F_1_ plant of the cross “4305” × “ZN198.” The ploidy level of all the regenerated plants achieved was estimated using an FCM Ploidy Analyzer (Partec GmbH, Germany) and only diploids were selected to construct the mapping population. Parents and DH lines were planted in the experiment field of Zhejiang Academy of Agricultural Sciences in Hangzhou, China and were preserved for long-term utilization by artificial selfing.

Young leaves were collected and genomic DNA was isolated according to a modified version of the cetyltrimethyl ammonium bromide (CTAB) procedure (Doyle and Doyle, 1986). DNA concentration and quality were detected using an ND-1000 spectrophotometer (NanoDrop, Wilmington, DE, USA) and electrophoresis on 1.0% agarose gel with a standard lambda DNA.

### SLAF library preparation and high throughput sequencing

A SLAF-seq strategy was used, as previously described by Sun X. et al. (2013), with modifications. First, reference genome of *B. oleracea* (cabbage, *B. oleracea* var. *capitata*, http://www.ocri-genomics.org/bolbase/, Liu S. et al., 2014) was used to design marker discovery experiments by simulating *in silico*, the number of markers produced by different enzymes. A SLAF pilot experiment was performed to determine the optimized enzymes and restriction fragment size, while the SLAF library was conducted based on the pre-designed scheme. Subsequently, the genomic DNA (2 μg) was digested with 3.6 units *Rsa*I (New England Biolabs, NEB, USA) in 20 μl volume containing 1 × NEB buffer at 37°C for 2 h, and then a single nucleotide (A) was added by using 6 units Klenow Fragment (3′ → 5′ exo^−^) (NEB) and 10 nmol dATP at 37°C for 1 h. Duplex tag-labeled sequencing adapters (PAGE-purified, Life Technologies, USA) were then ligated to the A-tailed fragments using T4 DNA ligase (NEB) by incubating overnight at 16°C, then 65°C for 20 min to heat deactivate the T4 ligase. Polymerase chain reaction (PCR) was performed in a 100 μl final volume reaction mixture, which contained diluted restriction-ligation DNA samples, PCR primers (forward sequence: 5′-AATGATACGGCGACCACCGA-3′, reverse sequence: 5′-CAAGCAGAAGACGGCATACG-3′), dNTP, MgCl_2_ and Q5® High-Fidelity DNA Polymerase (PAGE-purified, Life Technologies). The PCR cycles were 98°C for 3 min, 18 cycles of 98°C for 10 s, 65°C for 30 s, 72°C for 30 s, followed by an extension step of 5 min at 72°C before storage at 4°C. The amplification products were purified using Agencourt AMPure XP beads (Beckman Coulter, High Wycombe, UK) and pooled, followed by separation on 2% agarose gel electrophoresis. Fragments ranging from 244 to 314 bp (with indices and adaptors) in size were gel-purified using a QIAquick gel extraction kit (Qiagen, Hilden, Germany) and diluted for pair-end sequencing (125 bp at each end) using an Illumina HiSeq 2500 system (Illumina, Inc.; San Diego, CA, USA) at Beijing Biomarker Technologies Corporation, according to the manufacturer recommendations.

### Sequence data grouping and genotyping

SLAF marker identification and genotyping were performed as previously described by Sun X. et al. (2013) and Zhang et al. (2015). First, raw reads were demultiplexed to individuals according to the barcode sequences. The reads with quality scores < Q30 (a quality score of 30; indicating 0.1% chance of an error, and thus 99.9% confidence) were filtered out. After the barcodes and the terminal 5-bp positions were trimmed, high-quality reads from the same sample were mapped onto the reference genome sequence using SOAP software (Li et al., 2008). Subsequently, sequences locating at the same position with over 95% identity were grouped into one SLAF locus. SNP of each locus were firstly detected between parents, and SLAFs with less than three SNPs were used to define alleles. As cauliflower is diploid, only SLAFs with two to four alleles were identified as polymorphic and considered potential markers. Each polymorphic SLAF marker was then classified into eight segregation patterns in population (ab × cd, ef × eg, hk × hk, lm × ll, nn × np, aa × bb, ab × cc, and cc × ab) as previously described by Zhang et al. (2013). Since the DH mapping population used here was derived from two homozygous lines, only the SLAF markers showing aa × bb segregation pattern were used for map construction. Genotype scoring was then performed using a Bayesian approach to further ensure the genotyping quality, and high-quality SLAF markers for the genetic mapping were filtered by criteria as previously described by Sun X. et al. (2013).

### Genetic map construction

The genetic map was constructed as previously described by Zhang et al. (2015). Marker loci were allocated primarily into nine linkage groups (LGs) based on their locations on the reference genome. Markers with the modified logarithm of odds (MLOD) score < 5 were filtered to further confirm the marker robustness. A newly developed High Map strategy (Liu D. et al., 2014) was applied to order the SLAF markers and correct genotyping errors within LGs. MSTmap algorithm was used to order SLAFs markers (Wu et al., 2008) and the SMOOTH algorithm (Hans et al., 2005) was used to correct genotyping errors following marker ordering. Map distances were estimated using the Kosambi mapping function (Kosambi, 1943). Since all the mapped markers have been mapped onto the reference genome of *B. oleracea* by SOAP software (Li et al., 2008), the collinearity of physical map and genetic map were visualized by RSCRIPT language following the tutorial introduction.

## Results

### The mapping population

In total, 136 regenerated plants with different genotypes were obtained from “4305” × “ZN198” F_1_ by microspore culture. Several ploidy levels were detected in these plants, including haploids, diploids, polyploids, and chimeras (Figure S2). Finally, 79 diploid plants showing a spontaneously doubling ratio of 58.1% were obtained and used as the mapping population.

### SLAF sequencing and genotyping

**Figure 1:**
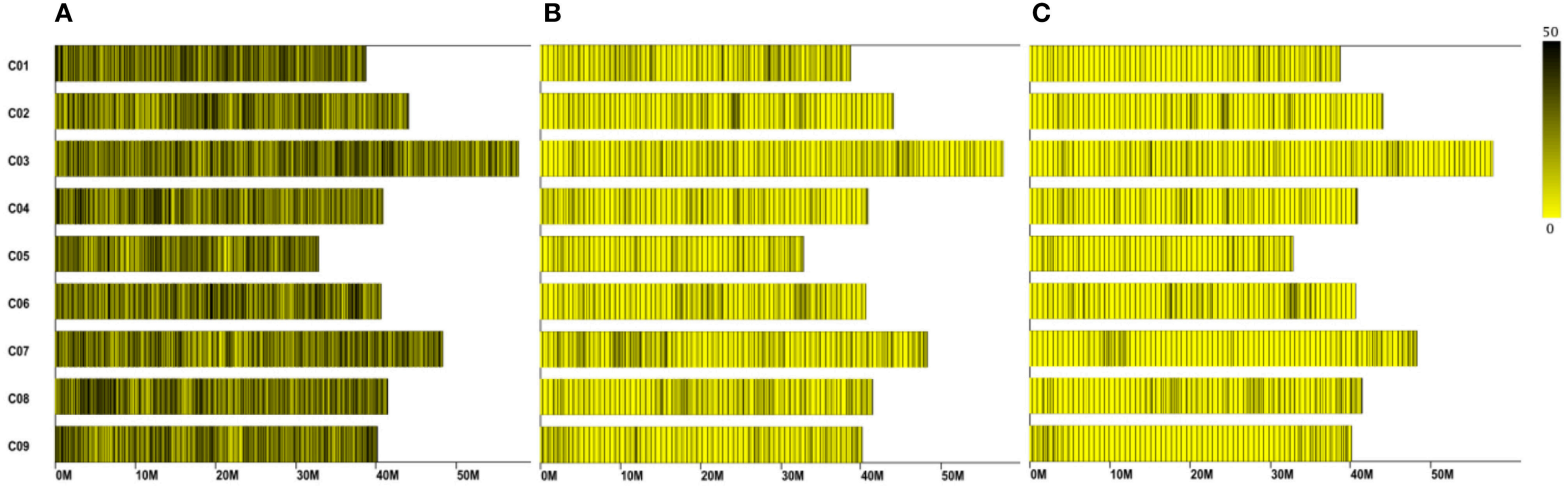
**Distribution of the total SLAFs (A) polymorphic SLAFs (B) and mapped SLAF markers (C) on the reference genome**. The x-axis represents chromosome length and the y-axis indicates chromosome code. Each yellow bar stands for a chromosome, and deeper color from yellow to black means more SLAFs on the corresponding location.

**Figure 2:**
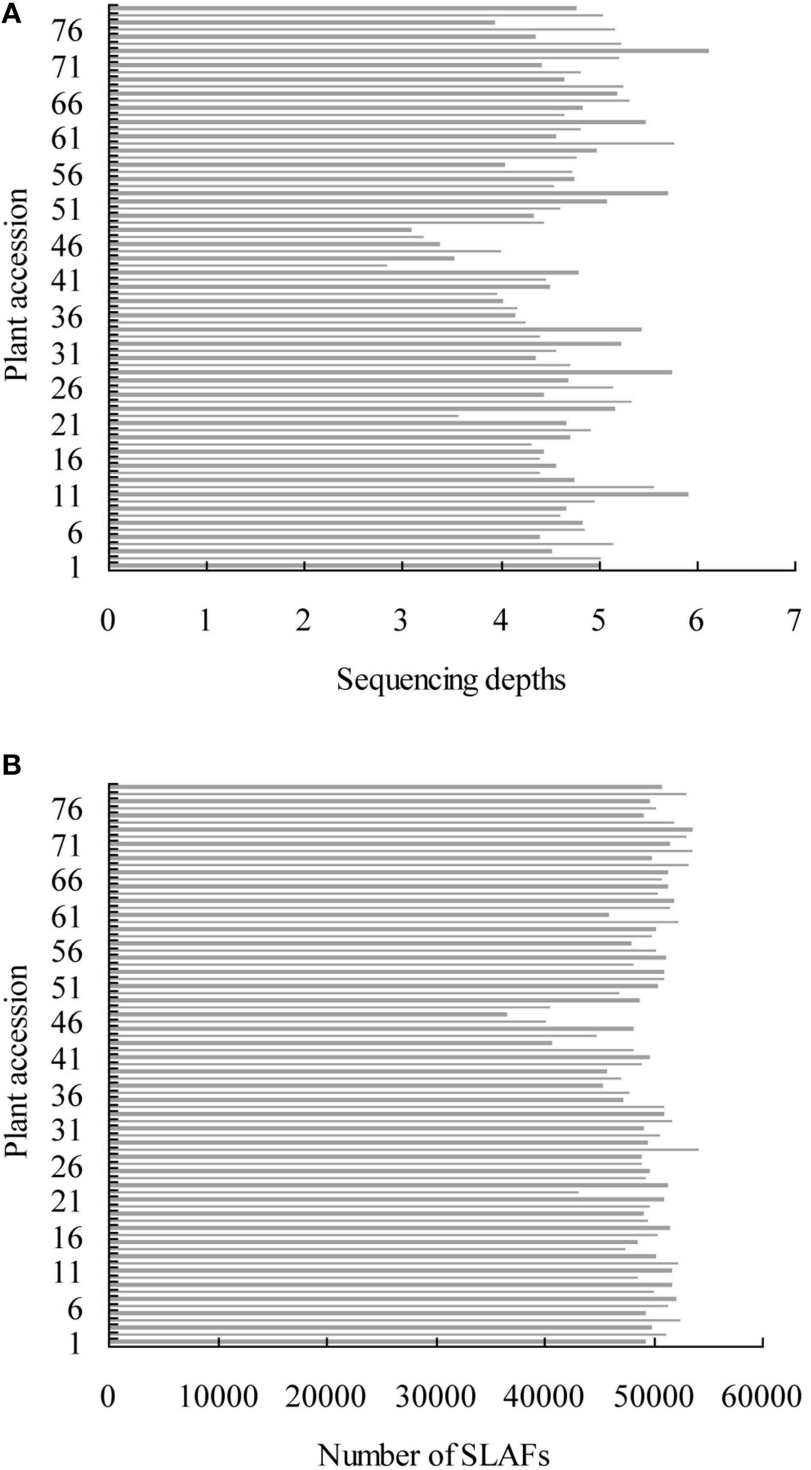
**Sequencing depths and number of SLAFs of all the individuals of mapping population**. The x-axis indicates the average depths **(A)** and number of SLAFs **(B)** the y-axis represents individual accessions.

A total of 12.47 Gb raw data containing 77.92 M pair-end reads were generated from the high-throughput sequencing (Data has been submitted to National Center of Biotechnology Information, the BioProject ID was PRJNA307521), with a GC (guanine-cytosine) content of 38.12%. The rate of high-quality reads with quality scores >30 reached 91.07%. After read clustering and filtering, 81,311 high-quality SLAFs with even distribution throughout the genome were identified (Figure 1A; Table 1; Table S1). The average sequencing depths of these SLAFs were 52.66-fold for female inbred line (4305), 49.35-fold for male DH line (ZN198), and 4.71-fold for each progeny of the DH population (Figure 2). Based on the allele number and sequence difference, the 81,311 SLAFs were grouped into three types including polymorphic, non-polymorphic, and repetitive. 6815 polymorphic SLAFs out of all SLAFs were obtained, showing a polymorphism rate of 8.38% (Figure 1B; Table S2). Interestingly, the polymorphism rate of SLAFs developed on each chromosome was quite different. In the current study, SLAFs on chromosome C01, C07, and C08 showed significantly higher polymorphism rate than the others (Table 2).

**Table 1:** **SLAF-seq data summary for the mapping population**.

**TOTAL READS**	
No. of reads	77.92 M
**HIGH QUALITY SLAFs**	
No. SLAFs	81,311
Average SLAF depth	6.33
Average depth in parents	51.01
Average depth in population individuals	4.71
**POLYMORPHIC SLAFs**	
No. of polymorphic SLAFs	6815
Average depth in parents	57.00
Average depth in population individuals	4.52
**HIGH-QUALITY SLAF MARKERS**	
No. of high-quality SLAF markers	1776
No. of SNPs	2741
Average depth in parents	61.40
Average depth in population individuals	4.50

**Table 2:** **Polymorphism rate of SLAFs developed on each chromosome**.

**Chromosome ID**	**Total SLAF**	**Polymorphic SLAF**	**Polymorphism rate %**
C01	8856	857	9.68
C02	8589	676	7.87
C03	12,049	898	7.45
C04	8779	654	7.45
C05	6918	453	6.55
C06	8797	726	8.25
C07	9892	1053	10.64
C08	9096	845	9.29
C09	8335	653	7.83
Total	81,311	6815	8.38

**Figure 3:**
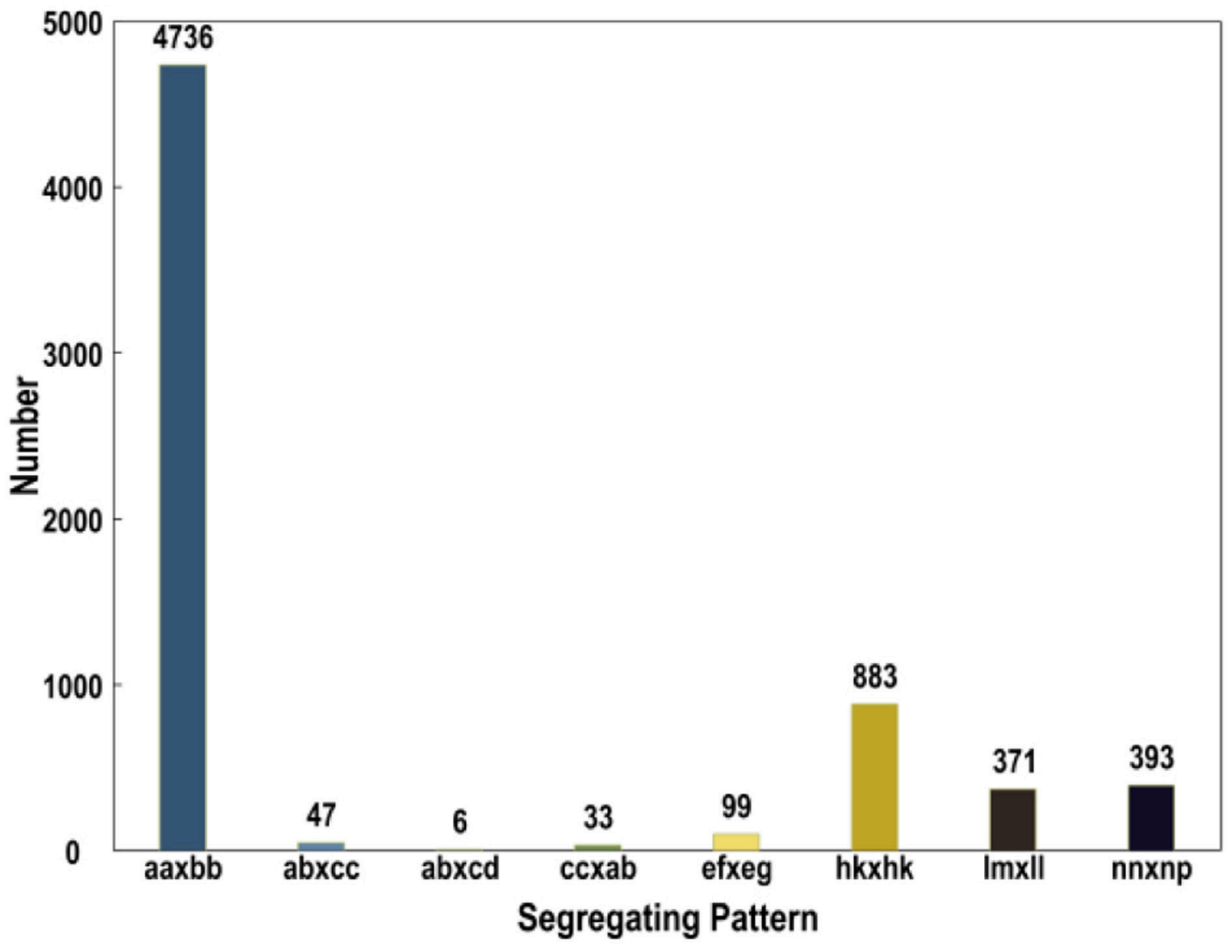
**Number of SLAF markers in each segregation patterns**.

Subsequently, all these polymorphic SLAFs were genotyped separately for parents and population individuals. A total of 6568 polymorphic SLAFs from total 6815 were successfully encoded, of which 4736 SLAFs were classified as the expected aa × bb segregation pattern, following the genotype encoding rule (Figure 3). These 4736 SLAFs were further filtered based on the criteria considering segregation distortion, sequencing depth, and data integrity. Finally, 1776 high-quality markers, with the average sequencing depths of 63.05-fold in the female parent, 59.75-fold in the male parent, and 4.50-fold in each DH individual, were employed to construct the genetic map (Figure 1C; Table 1).

### Main characteristics of the genetic map

**Figure 4:**
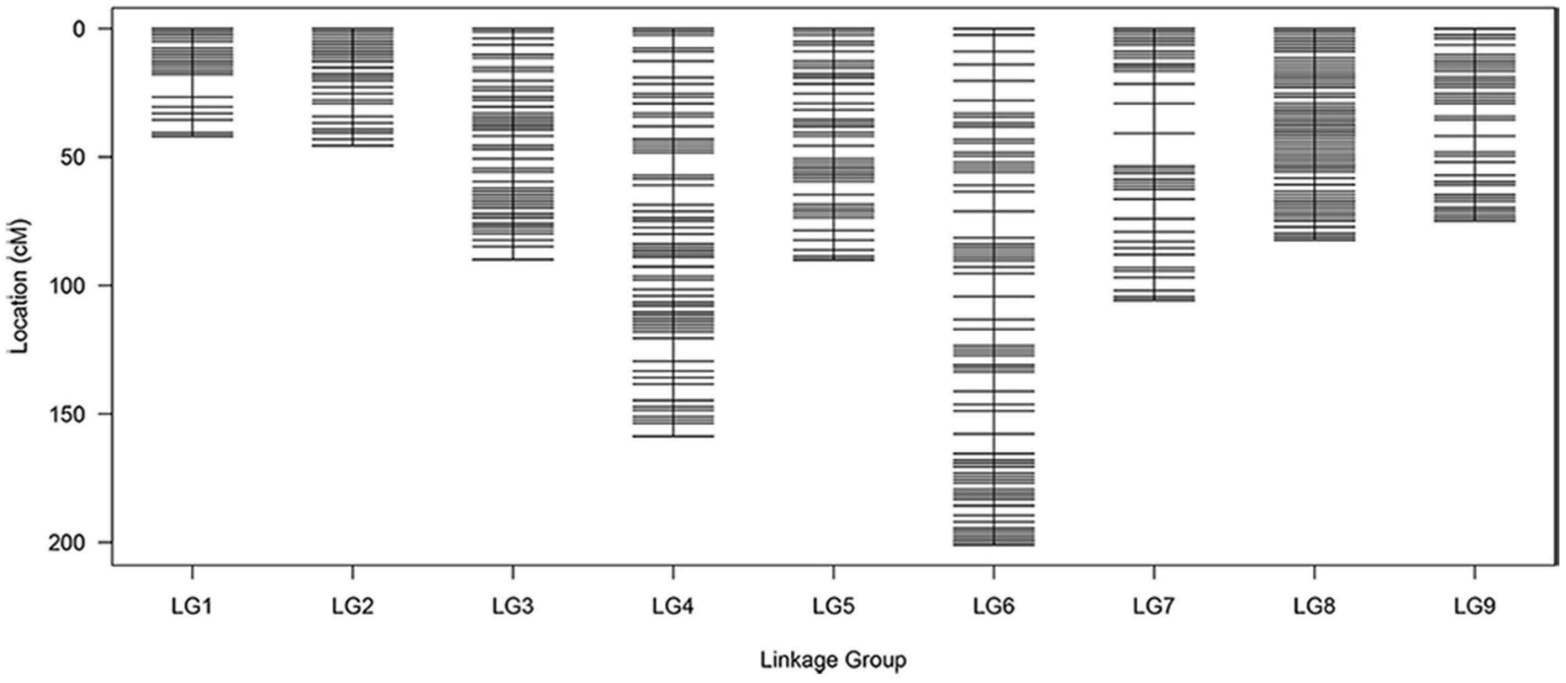
**Distribution of SLAF markers on eight linkage groups of cauliflower**. A black bar indicates a SLAF marker. The x-axis represents linkage group number and the y-axis indicates genetic distance.

All 1776 high-quality markers were successfully assigned onto 9 LGs according to their locations on the reference genome and the MLOD scores with other markers (at least one MLOD score >5). The average data integrity of these 1776 SLAF markers reached 95.68%. The final genetic map was constructed, following linkage analysis for each of the 9 LGs, which was designated according to the corresponding chromosome number of the reference genome (Figure 4; Table 3). A total of 1776 mapped SLAF markers containing 2741 SNPs spanned a total genetic length of 890.01 cM, with an average marker interval of 0.50 cM and covered 364.9 Mb of the reference genome. The genetic length, marker number, and average marker interval of single LG ranged from 41.90 (C01) to 200.92 (C06), 144 (C01) to 277 (C03), and 0.23 (C02) to 0.87 cM (C06), respectively. The max gap was 12.94 cM, located on C07. Detailed data of the genetic map and markers are presented in Table S3.

**Table 3:** **Basic characteristics of the nine LGs**.

**LG ID**	**Total SLAFs**	**Total SNPs**	**Size (cM)**	**Physical length (Mb)**	**Average Marker Interval (cM)**	**Gap ≤ 5 (%)**
C01	144	219	41.90	36.6	0.29	98.60
C02	199	291	45.61	41.7	0.23	99.49
C03	277	420	89.97	53.9	0.32	99.64
C04	213	320	158.70	39.7	0.75	96.23
C05	128	196	90.01	31.3	0.70	97.64
C06	232	355	200.92	37.1	0.87	93.07
C07	164	267	105.77	44.5	0.64	95.09
C08	274	439	82.31	40.6	0.30	100.00
C09	145	234	74.82	39.3	0.52	97.22
Total	1776	2741	890.01	364.9	0.50	98.60

“*Gap ≤ 5” indicates the percentage of gaps in which the distance between adjacent markers was smaller than 5 cM*.

### Visualization and evaluation of the genetic map

The mapped markers were anchored on the reference genome and the correlation of genetic and physical position is shown in Figure S4. Generally, a sufficient genome coverage and accurate genetic location of markers were revealed by the consecutive curves. The genetic arrangements of most markers were also considered to coincide with their physical direction based on the falling trend of the curve. However, a significant inversion can be observed intuitively on pseudo-chromosome 4. The detailed collinearity and marker location are described in Figure S3; Table S3.

Haplotype maps were generated for each DH individual with two parents as control using all the mapped markers in order to detect double recombination and deletion, thus to reflect potential genotyping and marker-order errors (West et al., 2006). In this study, there was no double recombination and deletion found in any linkage group (Figure S5). In addition, the map quality was also evaluated by heat maps, which could intuitively display recombination relationships among markers within each single LG. Pair-wise recombination rates could be visualized by different color levels from yellow to purple. Here, yellow color generally showed diagonal distribution in the heat map for each of the nine LGs (Figure S6), indicating that the mapped marker had been correctly ordered.

## Discussion

Due to the genome-wide abundance and high-throughput nature, SNP markers are playing an increasingly important role in plant research, such as genetic map construction, novel gene discovery, evolutionary analysis, and MAS within breeding programs (Liu et al., 2012). SLAF-seq is a newly reported enhanced RRL sequencing solution for large-scale SNP discovery and genotyping (Sun X. et al., 2013). It can produce large amounts of sequence-based information and handle any density distribution throughout the whole genome (Chen et al., 2013). Moreover, compared with another widely used, NGS-based method, RAD-seq, SLAF-seq shows higher reproducibility (Qi et al., 2014; Xu et al., 2014), and locus discrimination efficiency (Wei et al., 2014) due to its paired-ends sequencing strategy and longer read length (30–50 bp), respectively. In this study, we used this cost-effective strategy to develop and sequence a total of 81,311 SLAFs. Among these, 6815 SLAFs showing polymorphism between two parents (“4305” and “ZN198”) were identified with a polymorphic rate of 8.38%. Although the rate was much lower than that previously obtained by whole-genome re-sequencing in cabbage (Wang et al., 2012), the markers developed herein covered all pseudo-chromosomes and were evenly distributed throughout the reference genome (Figure 1B). In addition, the reads quality score of > 30 and an average sequencing depth of 57-fold for parents (Table 1) ensured high genotyping accuracy. Therefore, this set of SLAFs has great potential for use in both genomic study and breeding application of cauliflower. Additionally, our study further demonstrated that SLAF-seq is an efficient strategy for genome-wide SNP identification by sampling and sequencing a reduced set of representative genome regions instead of the whole genome.

Long-term domestication and selective breeding have resulted in abundant variations within *B. oleracea* species, hence several morphologically different subspecies currently available worldwide, such as cabbage, brussel sprouts, cauliflower, and broccoli (Kennard et al., 1994). In order to make the most of genetic variation within *B. oleracea*, traditional genomic researches usually employ different subspecies as parental materials to develop markers, construct genetic maps, and identify QTLs (Wang et al., 2012). However, these advances have been not comprehensive enough to support the genetic improvement of some specific organ, like head of cabbage or curd of cauliflower. For this reason, increasing attention has been given to genomic study based on intra-subspecies populations. For instance, Wang et al. (2012) developed more than 5000 SSR and SNP markers using two cabbage inbred lines and constructed a high-density genetic map including 1227 markers. Based on another cabbage × cabbage population, Lv et al. (2014) identified 707 InDels (insertion–deletions) and detected 13 reliable QTLs associated with five important heading traits. Among the *B. oleracea* varieties, cauliflower is the only plant where the immature inflorescence shows hypertrophic structure (curd). Therefore, the improvement of curd-specific traits like weight, size, shape, color, and content of nutritional compounds are important goals for the cauliflower breeding program. In this study, we used two different types of cauliflower, commonly produced in China, including an advanced inbred line of traditional compact-curd cauliflower and a DH line of loose-curd cauliflower, to identify SNPs and construct a genetic map. Firstly, the genetic base difference between loose-curd cauliflower and compact-curd cauliflower (Zhao et al., 2014b) would ensure acceptable marker polymorphism. Secondly, loose-curd cauliflower is now rapidly widespread and becoming a main cultivated type of cauliflower in China (Zhao et al., 2012). It shows varied agronomic characteristics (Zhao et al., 2013) and nutritional compounds (Gu et al., 2015) with a compact curd. The cauliflower-based SNP information should be helpful to enhance the future breeding program. In our lab, QTL mapping that aims to uncover the genetic factors controlling a series of curd-specific traits and to identify related markers based on the current study is now underway. In addition, loose-curd cauliflower originating from a portion of the genetic variation of domesticated cauliflower has undergone founder effects and intense selection for locally favorable curd characteristics (Zhao et al., 2014b). The significantly higher polymorphism percentage of SLAFs developed on C01, C07, and C08 between two parents suggest that these chromosomes may carry more genes/QTLs related to curd traits that have been strongly selected by breeders and cultivators. An interesting future task is to more thoroughly dissect the domestication of loose-curd cauliflower by using more abundant germplasm of both type of this crop.

Due to the limited genetic base within cauliflower (Zhao et al., 2014b), there has been very few genetic mapping studies using cauliflower based on intra-subspecies cross. The only case to our knowledge is a cauliflower × cauliflower-based map spanning a genetic length of 668.4 cM with 234 AFLP and 21 NBS (nucleotide binding site) markers (Gu et al., 2007). However, the limited marker numbers and density confined its further application. Besides, the markers in this map were difficult to be anchored onto the reference genome or transferred to other genetic maps. For all *B. oleracea* sub-species, the genetic map with highest marker density developed to date was based on *B. oleracea* Genome Sequencing Project (BrGSP) and whole-genome re-sequencing in cabbage. In this case, the map comprised 1227 markers, with an average interval of 0.98 cM, and was applied to anchor assembled scaffolds onto pseudo-chromosomes in BrGSP (Wang et al., 2012). In contrast, the SLAF-seq-based map in the present study contained 1776 SLAF markers including 2741 SNPs and covered 364.9 Mb of the cabbage genome. The marker quantity and resolution herein were significantly improved. However, many markers on the current map were noted to be highly clustered, some of them even located on the same locus, although their corresponding physical positions were quite different (Table S3). Therefore, despite the average marker interval being as short as 0.50 cM, there were still several obvious gaps on some regions. The similar phenomenon has been reported previously as well (Wang et al., 2012; Qi et al., 2014; Cai et al., 2015), indicating that the genetic differences or recombination between mapping parents on some genome regions were inadequate (Sun L. et al., 2013). In any case, the cluster markers may be separated distinctly if they are used for a different or broader population. For single LG, we also noted the discrepancy between the genetic length and corresponding pseudo-chromosome length of *B. oleracea* draft genome. For example, C06 is the longest LG in our map with a genetic length of 200.92 cM, but it is a relative short pseudo-chromosome in reference genome draft (http://www.ocri-genomics.org/bolbase/). This case, though common, could represent the extensive regions of increased/decreased recombination or could potentially indicate areas of the draft sequence that might require further refinement.

Comparative analyses of *Brassica* accessions have demonstrated that both genes relocation and sequence polymorphisms between species are common in the *Brassica* genome (Hu et al., 1998; Sebastian et al., 2000). Here, we show that the genome of cabbage and cauliflower are highly collinear, with macro collinearity punctuated by rearrangements mainly involving translocations and inversions. The majority of the observed rearrangements involved very short distances, but an apparent inversion spanning distance of more than 20 cM was noted at the central section of linkage group 04 between Marker13078 and Marker17566 (Figure S3; Table S3). Similar violation of genetic collinearity have also been identified within both intraspecific and interspecific level in other *Brassica* species, which may be responsible for the high degree of morphological polymorphism among these species (Sebastian et al., 2000; Bancroft et al., 2011). Since the cabbage and cauliflower subspecies shared a polyphyletic origin in primitive *B. oleracea* populations (Truco et al., 1996), our results imply that the inverted region seem to have been involved in the divergence of cauliflower and cabbage. Although additional genomic data from other varieties/lines are necessary to trace the genome evolution, comparative information about the collinearity between these two closely related subspecies has important implications for further marker and gene identification in cauliflower through the use of sequence data from cabbage as a genomic model.

In conclusion, we demonstrated the application of SLAF-seq strategy in cauliflower for large-scale SNP discovery and high-density genetic map construction. The parents used in this study are not only representatives of cultivated cauliflower, but also elite lines in our breeding program. Therefore, the genome-wide SNPs identified and the linkage map developed in this study will provide an important foundation not only for QTL identification and map-based cloning, especially of curd development-related traits, but also for MAS within cauliflower breeding programs.

## Author contributions

ZZ and HG conceived and performed experiments and wrote the manuscript; XS, HY, and JW were involved in data analysis; LH and DW performed the SLAF library construction and sequencing. All the authors have commented, read and approved the final manuscript.

## Funding

This project is supported by the National Natural Science Foundation of China (grant number, 31501768); the Research project in Zhejiang Province Science and Technology department (grant number, 2012C12903-3-4, 2016C32102); the Research project in Zhejiang Academy of Agricultural Sciences (grant number, 2014CX031, 2015R23R08E05).

### Conflict of interest statement

The authors declare that the research was conducted in the absence of any commercial or financial relationships that could be construed as a potential conflict of interest.
