# Supplementary figures and images for: Genome-Wide Single-Nucleotide Polymorphisms Discovery and High-Density Genetic Map Construction in Cauliflower Using Specific-Locus Amplified Fragment Sequencing
Source: Front Plant Sci. 2016 Mar 21;7:334. doi: 10.3389/fpls.2016.00334 (PMC4800193; doi:10.3389/fpls.2016.00334)

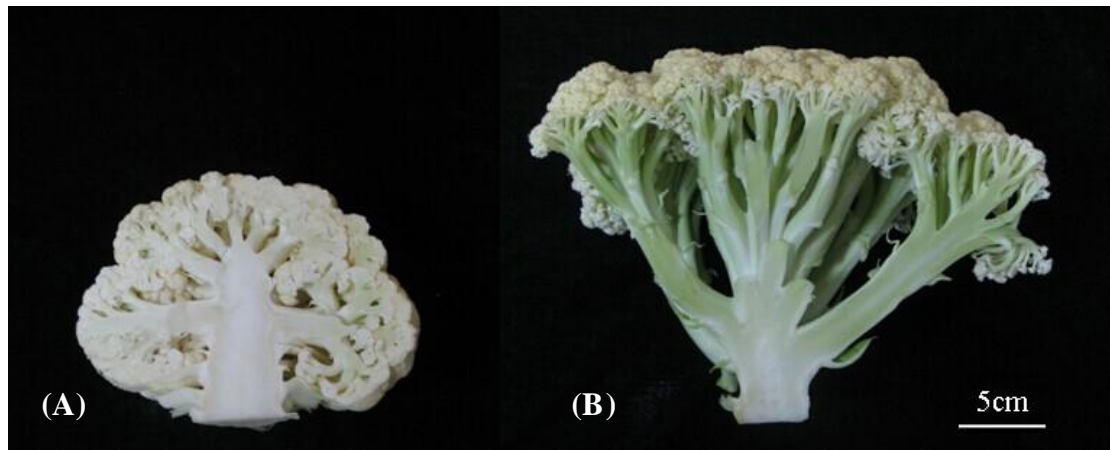

**Figure S1 | The curds of two parents used in the present study. A, ‘4305’; B, ‘ZN198’.**

Supplement: Supplementary file 4 [file Image1.PDF]
