# Supplementary material for: Genome-Wide Single-Nucleotide Polymorphisms Discovery and High-Density Genetic Map Construction in Cauliflower Using Specific-Locus Amplified Fragment Sequencing
Source: Front Plant Sci. 2016 Mar 21;7:334. doi: 10.3389/fpls.2016.00334 (PMC4800193; doi:10.3389/fpls.2016.00334)
Supplement: Supplementary file 5 [file Image2.PDF]

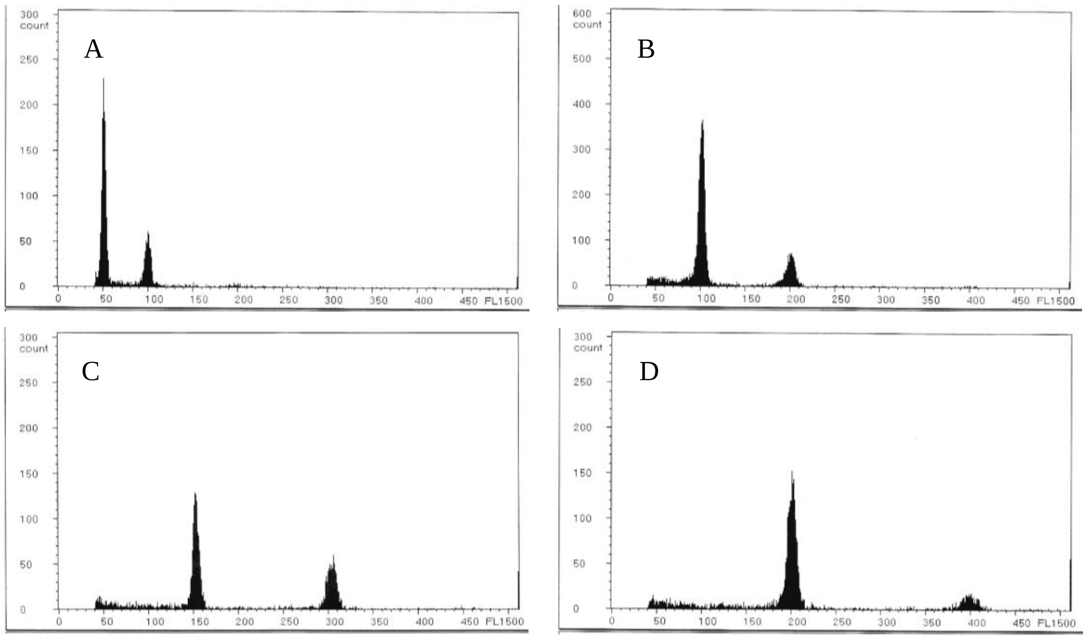

Figure S2 | Different ploidy levels of microspore culturing plants revealed by a FCM Ploidy Analyzer. A, haploid; B, diploid; C, triploid; D, tetraploid.
