# Supplementary material for: Genome-Wide Single-Nucleotide Polymorphisms Discovery and High-Density Genetic Map Construction in Cauliflower Using Specific-Locus Amplified Fragment Sequencing
Source: Front Plant Sci. 2016 Mar 21;7:334. doi: 10.3389/fpls.2016.00334 (PMC4800193; doi:10.3389/fpls.2016.00334)
Supplement: Supplementary file 6 [file Image3.PDF]

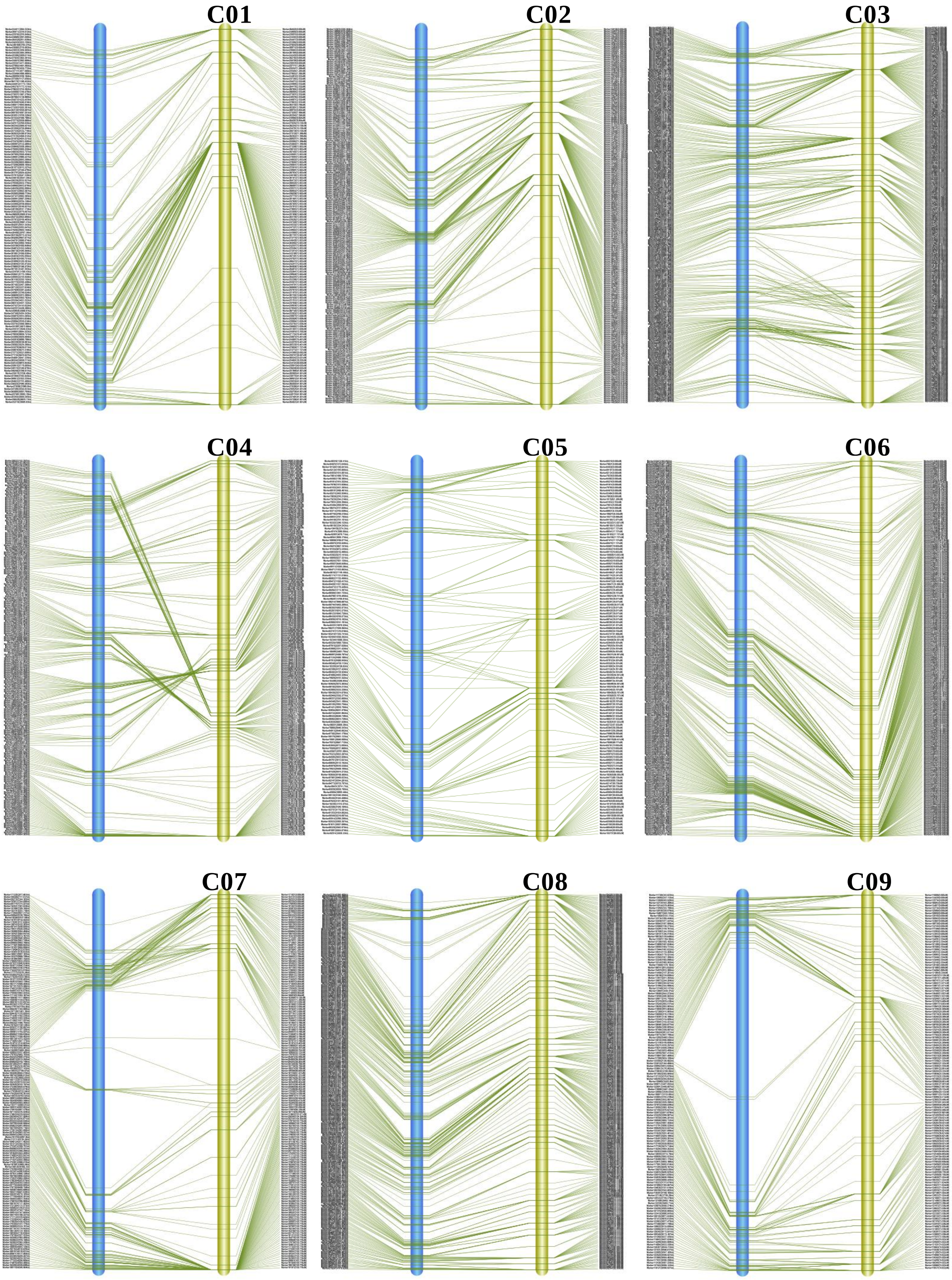

Figure S3 | High-density genetic map of cauliflower. The yellow columns represent linkage groups, and the blue columns represent chromosomes of reference genome. The green lines indicate the corresponding location on reference genome of mapping markers.
