# Supplementary material for: Genome-Wide Single-Nucleotide Polymorphisms Discovery and High-Density Genetic Map Construction in Cauliflower Using Specific-Locus Amplified Fragment Sequencing
Source: Front Plant Sci. 2016 Mar 21;7:334. doi: 10.3389/fpls.2016.00334 (PMC4800193; doi:10.3389/fpls.2016.00334)
Supplement: Supplementary file 7 [file Image4.PDF]

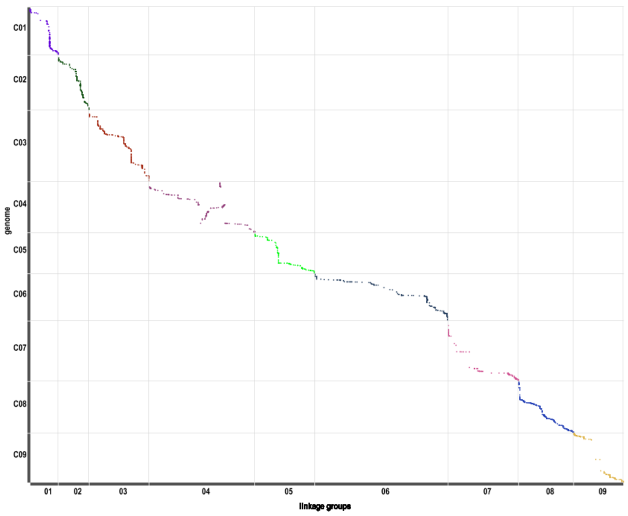

Figure S4 | Correlation of the genetic and physical positions. The x-axis represents the linkage group; the y-axis presents the physical positions of reference genome (cabbage).
