# Supplementary material for: Genome-Wide Single-Nucleotide Polymorphisms Discovery and High-Density Genetic Map Construction in Cauliflower Using Specific-Locus Amplified Fragment Sequencing
Source: Front Plant Sci. 2016 Mar 21;7:334. doi: 10.3389/fpls.2016.00334 (PMC4800193; doi:10.3389/fpls.2016.00334)
Supplement: Supplementary file 8 [file Image5.PDF]

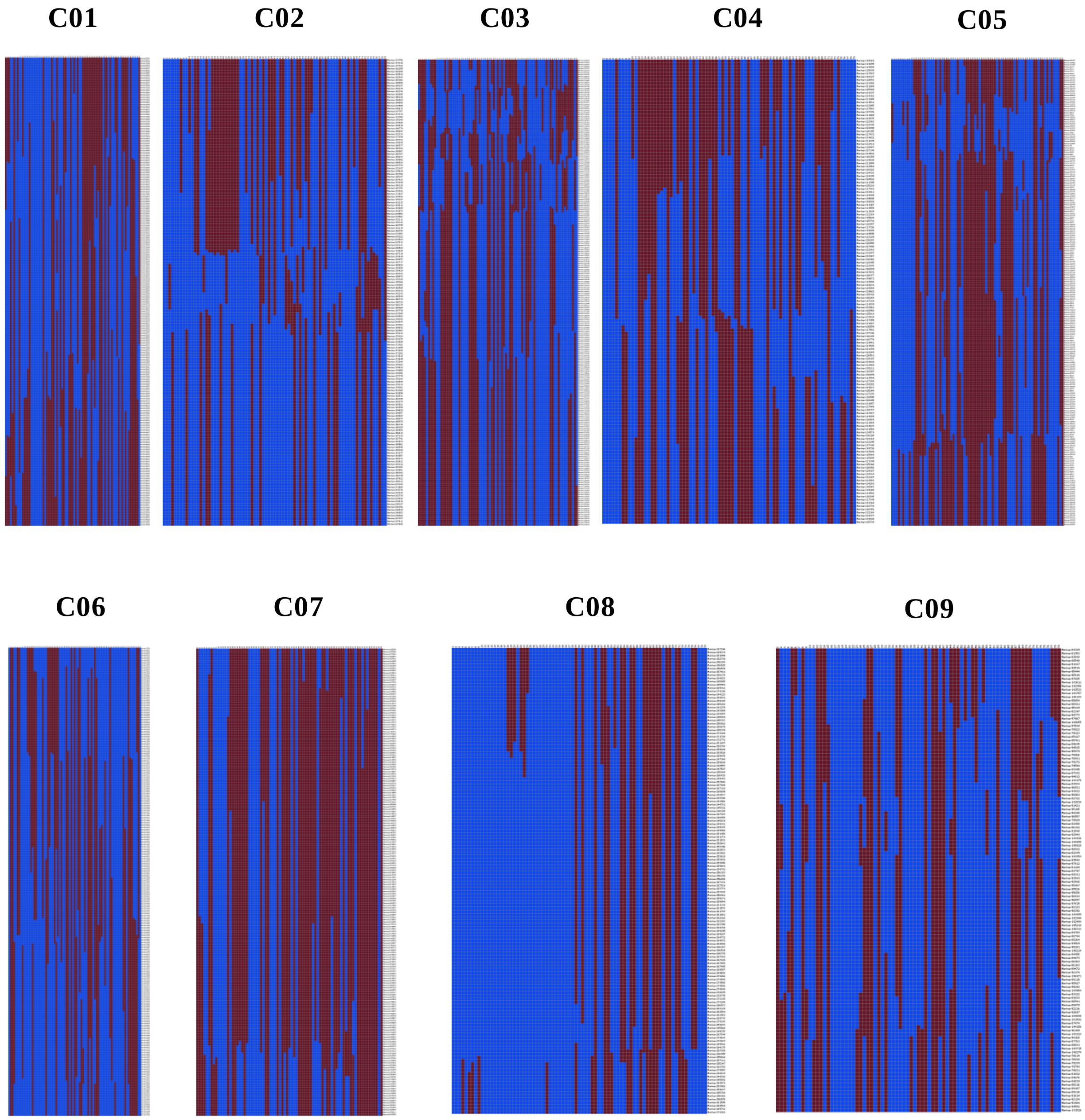

Figure S5 | Haplotype map of the genetic map. Blue represents 4305, red represents ZN198. Columns represent the genotype of an individual. Rows correspond to genetic markers. The position in each column where the color changed means there is recombination event.
