# Supplementary material for: Genome-Wide Single-Nucleotide Polymorphisms Discovery and High-Density Genetic Map Construction in Cauliflower Using Specific-Locus Amplified Fragment Sequencing
Source: Front Plant Sci. 2016 Mar 21;7:334. doi: 10.3389/fpls.2016.00334 (PMC4800193; doi:10.3389/fpls.2016.00334)
Supplement: Supplementary file 9 [file Image6.PDF]

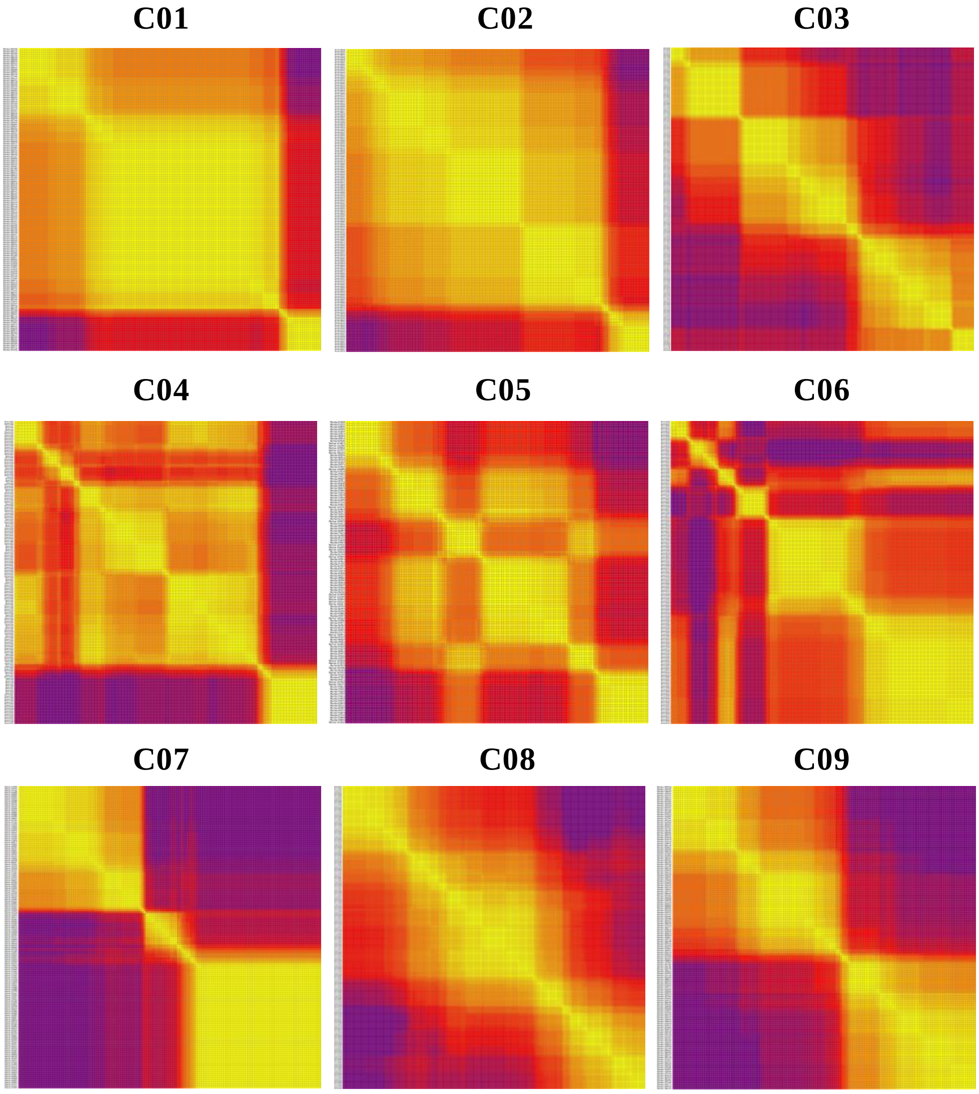

Figure S6 | Heat map of the genetic map. Each cell represents the recombination rate of two markers. Yellow indicates a lower recombination rate and purple indicates a higher one.
